# The brain’s sweet spot: why microglia can run on fructose metabolism

**DOI:** 10.3389/fimmu.2026.1864729

**Published:** 2026-07-06

**Authors:** Yasmina Eshac, Julieanne Cabang, Jason Miska

**Affiliations:** 1 Department of Neurological Surgery, Feinberg School of Medicine, Northwestern University, Chicago, IL, United States; 2 Malnati Brain Tumor Institute of the Robert H. Lurie Comprehensive Cancer Center, Feinberg School of Medicine, Northwestern University, Chicago, IL, United States; 3 Chicago Medical School, Rosalind Franklin University, North Chicago, IL, United States

**Keywords:** brain tumors, fructose, microglia, neurodevelopment, traumatic brain injury

## Abstract

Microglia are the resident myeloid cells of the central nervous system (CNS), and their identity and function are shaped by developmental origin, local signaling, and metabolic specialization. The fructose transporter GLUT5 (*SLC2A5*) stands out as a selective and conserved microglial marker that remains strongly associated with resident microglia across homeostatic and disrupted CNS states. In the CNS, fructose appears to be regulated locally rather than reflecting circulating levels, raising the possibility that fructose metabolism serves a unique functional role in microglia. Recent studies suggest that this pathway has a meaningful functional role. Fructose availability in the CNS can directly reshape microglial behavior by altering phagocytosis, redox balance, metabolic state, and downstream immune signaling in contexts ranging from neurodevelopment to glioblastoma. Our recent study showed that microglia fructose metabolism in glioblastoma drives an immunosuppressive state, limiting antigen presentation and downstream anti-tumor immunity. In this review, we examine microglial identity and metabolism in the context of GLUT5 and fructose uptake, summarize evidence that fructose acts as a context-dependent regulator of microglial function, and discuss how this may reflect broader metabolic strategies used by resident cells in distinct fluid and tissue barriers. We propose that microglial fructose metabolism is not simply an incidental feature of these cells, but a biologically meaningful feature of CNS physiology with important implications for development, injury, and disease, though the mechanistic basis of this relationship remains an active area of investigation.

## 1 Introduction

Microglia, the resident myeloid cells of the central nervous system (CNS), are distinct from peripheral macrophages due to their unique molecular signature, permanent residence, and capacity for self-renewal (1, 2). Unlike peripheral immune cells, microglia reside in a tightly regulated neural environment where their physiology is shaped by variable nutrient availability, neurotransmitters, and metabolic constraints (3, 4).

Previous work has provided some insights into these metabolic processes. For example, homeostatic microglia rely on oxidative phosphorylation, yet retain metabolic flexibility, including the ability to utilize alternative substrates such as glutamine via mammalian target of rapamycin (mTOR)-dependent pathways when glucose is scarce (5, 6). Microglia can also contribute to central and systemic metabolic homeostasis even in the absence of pathology. Recent work highlights how hypothalamic microglia engage in synaptic pruning to influence energy balance, thermoregulation, and circadian rhythms, emphasizing the complexity of their homeostatic roles (7, 8). However, the underlying cellular metabolic pathways that support these functions remain incompletely defined.

Among the alternative substrates available in the CNS, fructose has received comparatively little attention. As fructose levels in the CNS are significantly higher than in the periphery, and the dedicated fructose transporter GLUT5 is selectively expressed on microglia (9), it’s somewhat surprising how little we know about the role of fructose in microglia. Several recent high-profile studies have shed some light on this pathway, yet key questions remain unresolved. These include whether GLUT5 represents a functional necessity or simply a lineage marker, how locally produced CNS fructose relates to microglial behavior, and what the biochemical consequences of fructose metabolism are in microglia specifically. The goals of this review are to provide an overview of microglial identity and metabolism in the context of fructose, to delineate what is established from what remains speculative, and to address why this axis even exists in the brain.

## 2 Developmental and lineage context

Microglia are CNS-resident myeloid cells that comprise developmentally distinct populations whose origins fundamentally shape their identity and function. Microglia arise from primitive hematopoiesis in the embryonic yolk sac rather than from bone marrow-derived hematopoietic stem cells. Fate-mapping studies demonstrated that yolk sac-derived erythromyeloid progenitors migrate into the developing neuroepithelium early in embryogenesis and give rise to microglia, which persist into adulthood through local self-renewal (10, 11). In contrast, bone marrow-derived macrophages arise from hematopoiesis and are delivered into the bloodstream as circulating monocytes. In steady-state conditions, bone marrow-derived macrophages are largely excluded from the CNS parenchyma, entering primarily during injury or inflammation (12).

Characteristic markers associated with CNS surveillance and homeostasis, including *Tmem119, P2ry12*, and *Sall1*, are found in microglia and help distinguish them from other myeloid and peripheral cells under homeostatic conditions (2, 13). Epigenetic studies further suggest that the structural organization of microglial chromatin differs from that of peripheral macrophages and does not depend on bone marrow replacement for maintenance. These findings indicate that developmental origin plays a major role in establishing microglial identity (14).

Beyond transcriptional identity, developmental origin also influences functional specialization within the CNS. Yolk sac-derived microglia perform several long-term homeostatic functions in the CNS, such as synaptic pruning, clearance of apoptotic cells and other cellular debris, and neuronal circuit maturation (15, 16), while infiltrating macrophages that are recruited during injury or disease often exhibit a more pro-inflammatory phenotype, increased cytokine production, and antigen presentation (17).

Homeostatic microglia primarily engage in oxidative phosphorylation consistent with their surveillance role (8). They also require increased lipid metabolism to support functions such as phagocytosis (18). By comparison, inflammatory macrophages often increase glucose uptake and rely more heavily on aerobic glycolysis during activation (19). These metabolic features of microglia align with their role as long-lived, self-renewing cells operating within a nutrient-variable CNS environment. Although environmental cues within the CNS influence cellular metabolism, developmental origin appears to contribute to baseline metabolic differences between resident microglia and infiltrating macrophages.

## 3 The physiological role of microglia is tied to their unique developmental identity

In the healthy brain, microglia continuously survey the parenchyma by extending and retracting processes and engage in phagocytosis without triggering overt inflammation. Microglial process dynamics are influenced by neuronal activity. Pharmacological inhibition and sensory deprivation suppress neuronal network activity, increasing microglial surveillance, whereas increased neuronal activity decreases process extension via norepinephrine-dependent signaling (20). These findings demonstrate that microglia dynamically respond to neural circuit activity and do not exist in a simple “resting” versus “activated” state.

Most of what is known about microglial function comes from pathological contexts, including neurodegeneration, infection, and traumatic injury. In these settings, microglia are involved in disease-associated processes, such as expression of major histocompatibility complex (MHC) II antigens in multiple sclerosis and their contribution to Alzheimer’s disease pathology (21). However, many of these conclusions are based on perturbation models, making it difficult to define a single physiological function that uniquely requires microglia under homeostatic conditions.

Ablation studies complicate the role and indispensability of microglia. Pharmacologic inhibition of the colony-stimulating factor 1 receptor (CSF1R) results in microglial depletion in adult mice (22). Despite this depletion, animals display relatively mild behavioral and structural abnormalities under baseline conditions. After withdrawal of CSF1R inhibition, the CNS can be repopulated by residual microglia. In some models, peripheral monocytes or macrophages can engraft into the CNS and adopt microglia-like morphology (23, 24).

These findings suggest that certain functions traditionally attributed to microglia, such as debris clearance and immune surveillance, may not be strictly exclusive to yolk sac-derived cells. However, repopulating macrophages does not fully replicate the molecular identity of embryonically derived microglia. Transcriptomic and epigenetic analyses reveal persistent differences in gene expression, chromatin accessibility, and metabolic programming between resident microglia and macrophage-derived replacements (13, 25). These non-replaceable features suggest that certain microglial properties are intrinsically specified by developmental origin rather than solely by the CNS environment. Identifying these intrinsic properties, including GLUT5-linked fructose metabolism, longevity, and interaction with neural circuits, may be key to defining the fundamental physiological role of microglia.

## 4 A specific link between microglia and fructose transport

Microglia retain a unique identity, influenced by their developmental origin and consistently reinforced by signals within the CNS. The field of immunometabolism has shown that nutrient transporters can serve as indicators of both lineage and function. GLUT5 (*SLC2A5*) is a facilitative fructose transporter that has emerged as a consistent and selective feature of microglia (26–28). Early protein-level studies showed that GLUT5 is highly expressed in microglia in both human and rodent brain tissue, with minimal detection in neurons, astrocytes, or oligodendrocytes (26). More recent transcriptomic and lineage-based datasets suggest this is not coincidental: *Slc2a5* consistently aligns with core microglial identity programs, including *Sall1*, in datasets that distinguish resident microglia from monocyte-derived cells. This is particularly evident in depletion-repopulation models: Lund et al. showed that CX3CR1+F4/80^lo^ yolk-sac-derived microglia retained *Sall1* and *Slc2a5*, while monocyte-derived CX3CR1+F4/80^hi^ cells failed to adopt this profile (29). Independent analyses further identify *Sall1* as a master regulator of microglial identity (30) and *Slc2a5* among the genes that most reliably distinguish resident microglia from other myeloid populations (31). These findings support GLUT5 as more than a useful marker; it is tightly linked to the transcriptional program that defines resident microglia.

An important question is whether the microglia-GLUT5 association is limited to homeostatic microglia, or if it persists when the CNS environment is disrupted by tumor, inflammation, or injury. Evidence from glioma studies supports the latter. In human glioma tissue, GLUT5-positive cells were localized to microglial populations rather than tumor cells, supporting GLUT5 as a microglial marker even in a remodeled microenvironment (28). More recent single-cell studies in glioblastoma show that resident microglia remain transcriptionally distinct from infiltrating monocyte-derived macrophages, indicating that microglia-specific programs can persist despite significant tumor-associated remodeling (32, 33).

A similar pattern is observed in inflammatory settings. During virus-induced neuroinflammation, which recruits infiltrating macrophages, microglia maintain distinct transcriptional profiles and retain microglia-specific marker patterns (34). This suggests that disruption of the CNS environment does not erase the molecular features that distinguish resident microglia. Taken together, these findings support the broad conclusion that GLUT5 is not simply a marker of homeostatic microglia but also remains preferentially associated with resident microglia across disrupted CNS contexts.

## 5 What makes fructose metabolism unique?

### 5.1 The biochemistry of fructose metabolism in microglia

Dietary fructose is absorbed from the intestinal lumen primarily via the GLUT5 transporter encoded by the SLC2A5 gene, which is highly specific for fructose (35). Once in the liver, fructose is phosphorylated by ketohexokinase (KHK) also known as fructokinase, to generate fructose−1−phosphate (F1P), bypassing the low-affinity phosphorylation of fructose by Fructokinase. F1P is then cleaved by Aldolase B into dihydroxyacetone phosphate (DHAP) and glyceraldehyde (GA), and GA is subsequently phosphorylated by triose kinase to form glyceraldehyde−3−phosphate (G3P), which feeds into glycolytic and gluconeogenic pathways (35, 36). Because fructose enters metabolism downstream of the rate-limiting enzyme phosphofructokinase-1 (PFK1), it largely bypasses the regulation that integrates cellular energy status and demand. This unregulated flux allows fructose-derived carbons to rapidly enter glycolytic intermediates and provides a carbon shunt into energetic pathways independent of the cell’s energetic state (35, 37).

In immune cells, including microglia, glycolytic regulation at PFK1 is a key feature of the activated state, with inflammatory microglia exhibiting a shift toward aerobic glycolysis that supports cytokine and reactive oxygen species (ROS) production. This is consistent with disease-associated microglial programs observed in neurodegeneration (38–40). Because of this glycolytic metabolism, there is a downstream accumulation of the glycolytic end-product lactate. Zhang et al. showed that in macrophages, lactate drives histone lactylation; therefore, coupling metabolic states to transcriptional output (41). This mechanism has also been demonstrated in microglia across multiple CNS disease contexts. For instance, glycolysis-derived lactate promotes pro-inflammatory gene transcription by driving H3K9 lactylation in microglia in Parkinson’s disease (42). Similarly, glycolytically reprogrammed microglia and macrophages utilize histone lactylation to drive chemokine transcription following spinal cord injury (43). These findings raise the possibility that fructose-derived lactate accumulation may represent an additional route through which microglial programs are epigenetically influenced in neurodegenerative contexts.

In KHK-expressing cells such as the liver, rapid fructose phosphorylation can transiently deplete adenosine triphosphate (ATP), thereby increasing nucleotide turnover and uric acid production and contributing to oxidative stress (37, 44). In microglia specifically, metabolic stress and ROS accumulation are linked to inflammatory signaling pathways, by activating Nuclear Factor Kappa B (NF-κB) and nucleotide-binding domain, leucine-rich-containing family, pyrin domain-containing-3 (NLRP3) inflammasome signaling, which drives the pro-inflammatory characteristic of disease-associated microglial states and contributes to pathogenesis in models of Alzheimer’s disease (AD) (39, 45).

Simultaneously, the accumulation of triose phosphate intermediates diverts carbon flux toward *de novo* lipogenesis and other biosynthetic pathways, including increased methylglyoxal production and, consequently, protein damage. This shift in lipid metabolism promotes the formation of intracellular lipid droplets, which can drive microglia toward the lipid-droplet-accumulating microglia (LDAM) phenotype, characterized by heightened oxidative stress and impaired phagocytic capacity (39, 46).

In contrast, extrahepatic fructose metabolism can proceed via hexokinases (HK), particularly HK2, which phosphorylates fructose to fructose-6-phosphate (F6P) and allows it to enter glycolysis. Fructose is thought to be slowly metabolized via HK2 and, in some contexts, may act more as a signaling molecule (47, 48). Despite this slower metabolism, microglia likely utilize both HK2 and KHK, with HK2 potentially predominating under physiological conditions and KHK playing a more prominent role in specific disease states.

**Figure 1:**
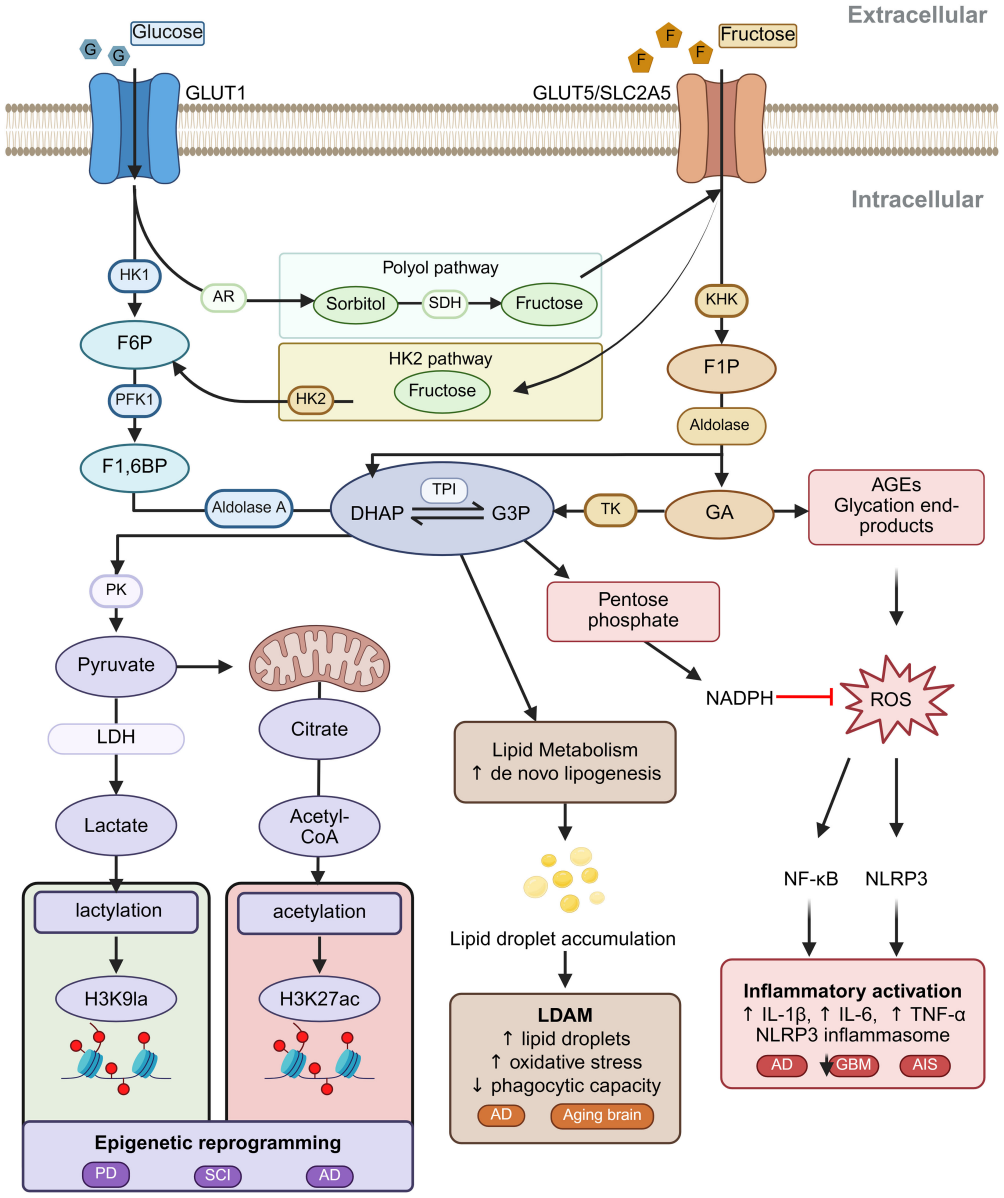
Fructose metabolism in microglia drives immunometabolic pathways implicated in CNS pathology. Unlike glucose, which is subject to rate-limiting regulation at PFK1, fructose bypasses this checkpoint via KHK, allowing unregulated carbon flux into glycolytic intermediates. This drives three downstream consequences with pathological relevance: lactate dehydrogenase (LDH)-mediated lactate production that promotes histone lactylation and acetylation, and epigenetic reprogramming of microglial states; lipid droplet accumulation leading to the dysfunctional LDAM phenotype; and AGE- and ROS-mediated activation of NF-κB and the NLRP3 inflammasome, inducing pro-inflammatory cytokine production. Together, these pathways implicate fructose as a significant immunometabolic substrate that can sustain pathological microglial states found in diverse CNS conditions including AD, PD, spinal cord injury (SCI), GBM, and acute ischemic stroke (AIS). AIS, acute ischemic stroke; AR, aldose reductase; Acetyl-CoA, acetyl coenzyme A; F1,6BP, fructose-1,6-bisphosphate; F6P, fructose-6-phosphate; H3K9la, histone H3 lysine 9 lactylation; H3K27ac, histone H3 lysine 27 acetylation; IL-1β, interleukin-1 beta; IL-6, interleukin-6; LDH, lactate dehydrogenase; NADPH, nicotinamide adenine dinucleotide phosphate; PK, pyruvate kinase; SCI, spinal cord injury; SDH, sorbitol dehydrogenase; TK, triose kinase; TNF-α, tumor necrosis factor alpha; TPI, triose phosphate isomerase.

Beyond enzymatic metabolism, fructose-derived intermediates from KHK metabolism, such as DHAP and GA are highly reactive chemically and prone to non-enzymatic glycation via Maillard reactions, forming reactive oxygen species (ROS) and advanced glycation end products (AGEs) that can impair protein function, disturb redox homeostasis, and affect signaling. These combined features of rapid, unregulated entry into glycolysis, ATP/AMP disruption, substrate flux toward lipogenesis, and chemical reactivity link fructose metabolism to significant alterations in metabolic networks and stress responses that engage the signaling pathways and phenotypic programs that may influence pathological microglial states across diverse CNS pathologies (**Figure 1**) (39, 44, 46, 49, 50).

### 5.2 Systemic fructose availability is decoupled from interstitial levels

This is especially important because fructose in the CNS should not be viewed as a reflection of systemic fructose metabolism. Although fructose is strongly linked to peripheral metabolic disease and inflammation (35, 51), the brain is also thought to generate fructose locally from glucose through the polyol pathway, as directly demonstrated by magnetic resonance spectroscopy that shows intracerebral fructose levels rising in response to changes in brain glucose independently of plasma fructose levels (52). Additionally, fructose concentrations are approximately 20-fold higher in cerebrospinal fluid (CSF) than in plasma, indicating that fructose availability in the CNS is not necessarily proportional to circulating levels (53). It should be noted, however, that CSF fructose levels are a surrogate rather than a direct measure of fructose in the parenchymal interstitium, where most microglia reside, and the spatial distribution of fructose across CNS compartments and cell types remains unresolved. This supports the argument that microglial fructose metabolism should be interpreted within a specific CNS metabolic context, in which fructose is locally produced, and microglia are uniquely equipped to take it up via GLUT5.

Glucose availability to non-neuronal cells in the brain is often overestimated. The majority of microglia are not located directly at the blood-brain barrier. Only a subset of microglia are in contact with vessels, reflecting a specialized perivascular niche rather than the typical microglial position in tissue (54). Further, electron microscopy supports the notion that only a fraction of microglia make direct contact with blood vessels, indicating that many microglia reside deeper in the parenchyma, away from the vascular interface (55). Since blood vessels are the primary entry point for nutrients such as glucose into the brain, microglia residing away from vessels may not have access to the highest local nutrient concentrations (55). It is therefore important to understand which metabolites are actually present in the extracellular space where microglia reside.

In humans, CSF glucose levels are approximately 60% of blood levels and are typically in the lower millimolar range, with age-specific 5th-95th percentile values of 1.8-5.6mmol/L (56). Conversely, several human cerebral microdialysis studies report extracellular brain glucose concentrations in the single-millimolar range, typically 0.8–1.7 mmol/L across perioperative and baseline conditions (57). This data argues against the common assumption that non-neuronal cells in the CNS are uniformly exposed to abundant glucose.

**Figure 2:**
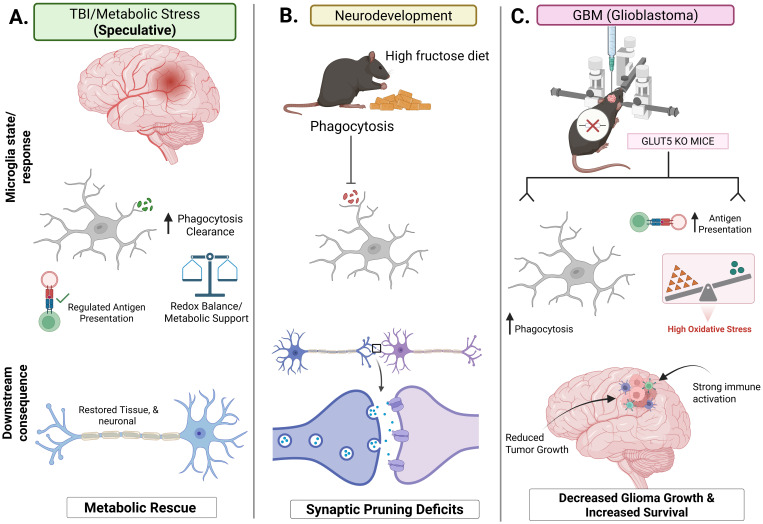
Microglial fructose metabolism exerts profound biological effects in the brain. In **(A)** based on the limited amount of glucose within the interstitium of the brain, which has been shown to be depleted under conditions of traumatic brain injury (TBI) (58, 59), we propose that the abundance of interstitial fructose acts as an alternative fuel source to maintain microglial function. In **(B)** a recent mechanistic study found that early-life high fructose consumption impairs microglial synaptic pruning by inhibiting phagocytosis (67). This provides a mechanistic understanding of the neurodevelopmental defects observed in high-fructose consumption models. In **(C)** our recent publication, we identified that mice deficient in GLUT5 exhibit dramatic tumor rejection across several glioma models (69). Mechanistically, we found that microglia deficient in GLUT5 exhibit increased antigen presentation and phagocytosis, which may be regulated by increased redox stress.

This becomes even more relevant under metabolic stress. In severe traumatic brain injury, cerebral microdialysis studies show that extracellular brain glucose can drop to <0.2 mmol/L in regions of high glucose utilization (58). During the first week after injury, glucose levels below 0.2 mmol/L were observed for approximately 1/5 to 1/3 of the monitored hours each day. These persistently low glucose concentrations correlated with worse long-term neurological outcomes. Neurocritical care studies show that brain glucose can fall to very low levels even when blood glucose is kept at a normal range, and low brain glucose together with a high lactate/pyruvate ratio is commonly used to define “brain energy crisis” after severe injury (59). These findings suggest that fructose availability may be most relevant specifically when microglia face metabolic stress, rather than under baseline conditions of high glucose (**Figure 2A**).

## 6 What is the role of microglial fructose metabolism in different pathologies?

With GLUT5 established as a feature of resident microglia, the next question is what fructose is doing mechanistically in microglia. While much is unknown, several studies provide correlative and mechanistic insights into how fructose availability may shape microglial behavior and function (60). **Table 1** summarizes the original studies examining microglial fructose metabolism across *in vitro* and *in vivo* contexts, distinguishing homeostatic from disease settings.

**Table 1:** Summary of original studies on microglial fructose metabolism.

Disease / context	Study	Model	*Ex vivo* / *In vivo* / *In vitro*	Key findings	Ref
Homeostatic	Payne et al. (1997)	Human and rat brain tissue	*Ex vivo*	GLUT5 is highly expressed in microglia with minimal expression in other brain cell types	(26)
Homeostatic	Lund et al. (2018)	Mice microglia depletion and repopulation models	*In vivo*	Only yolk sac-derived microglia retain GLUT5 expression; monocyte-derived replacements do not	(29)
Inflammatory Activation	Shen et al. (2025)	RAW 264.7 macrophage cell line; IMG immortalized microglial cell line; CRISPR-Cas9 GHSR knockout	*In vivo* and *In vitro*	Fructose directly activates inflammatory signaling in microglia through the nutrient-sensing ghrelin receptor (GHSR)	(62)
Neurodevelopment	Wang et al. (2025)	Neonatal C57BL/6 mice; primary mouse and human microglia	*In vitro* + *In vivo*	Fructose suppresses microglial phagocytosis via GLUT5; removing GLUT5 restores phagocytosis; HK2 mitochondrial relocalization identified as mechanism	(67)
Diabetes	Li et al. (2023)	Leptin receptor-deficient T2DM (db/db) mice; KHK knockout mice	*In vivo*	KHK is localized in microglia and upregulated in hippocampus of diabetic mice. Inhibiting KHK reduces microglial activation and improves cognition	(68)
Glioblastoma	Billingham et al. (2026)	RCAS-derived tumor models; GLUT5-deficient mice	*In vitro* + *In vivo*	GLUT5 loss reduces tumor growth and enhances anti-tumor immune responses; survival benefit depends on CD8+ T cells	(69)
Alzheimer's Disease (AD)	Marino et al. (2025)	5XFAD mice (Murine AD model) mice; N9 mouse microglial cell line	*In vitro* + *In vivo*	GLUT5 expression decreases in AD mouse models; fructose-fed microglia show a dampened response to amyloid pathology vs. glucose-fed microglia	(71)
Alzheimer's Disease (AD)	Leng et al. (2022)	5XFAD x CX3CR1-CreER HK2 conditional knockout mice; human AD brain tissue	*In vivo*	HK2 deletion in microglia promotes amyloid clearance and reduces cognitive impairment; fructose-6-phosphate reverses these effects	(72)
Ischemic Stroke	Ai et al. (2026)	Murine ischemic stroke model (AIS)	*In vivo*	GLUT5 loss reduces brain injury after stroke; reduces PKM2-driven pro-inflammatory reprogramming; shifts microglia toward a neuroprotective state	(73)

Studies are organized by disease context and experimental approach (*in vitro*, *in vivo*, or *ex vivo*). GLUT5, fructose transporter 5; HK2, hexokinase; KHK, ketohexokinase; PKM2, pyruvate kinase M2; GHSR, ghrelin receptor; GBM, glioblastoma; AD, Alzheimer’s Disease; AIS, acute ischemic stroke; T2DM, type 2 diabetes mellitus; 5XFAD, five familial Alzheimer’s disease mutations transgenic mouse model.

Previous studies have clearly identified that dietary fructose can induce maladaptive responses in the brain. In rodent models, high fructose has been linked to neuroinflammation, oxidative stress, mitochondrial dysfunction, and impaired synaptic plasticity and cognition, especially in brain regions involved in learning and memory (61). Fructose has been shown to directly induce microglial inflammation through the nutrient-sensing ghrelin receptor (GHSR), triggering downstream proinflammatory signaling (62). Other studies have further highlighted that chronic high-fructose intake disrupts brain insulin signaling and is linked to deficits in learning and memory across key developmental stages (63). In a conceptual review, Johnson et al. proposed that intracerebral fructose metabolism may contribute to neurodegenerative pathology, including Alzheimer’s disease (AD) (64, 65). Prospective human data have similarly linked fructose consumption to increased risk of all-cause dementia and Alzheimer’s disease (66). Most importantly, these studies examined systemic dietary fructose exposure rather than intrinsic microglial fructose metabolism. The findings reflect whole-brain responses to excess fructose intake rather than the locally produced fructose that microglia access via GLUT5. We include these studies here to provide a broader context for why brain fructose matters, not as direct mechanistic evidence for microglial function.

Connecting these broader observations to mechanisms, the Perry lab has recently provided direct evidence that fructose availability can shape microglial behavior by identifying microglia as key consumers of excess dietary fructose (67). In their study, early-life fructose exposure suppressed microglial phagocytosis *in vivo*, and high fructose similarly impaired phagocytosis in both mouse and human iPSC microglia, and depletion of GLUT5 in neonatal microglia rescued the phagocytic abilities. Mechanistically, microglia took up fructose via GLUT5 and catabolized it to fructose 6-phosphate, rewiring microglial metabolism toward a hypophagocytic state, in part by promoting mitochondrial localization of hexokinase 2 (HK2), a key glycolytic enzyme. *In vivo*, this was accompanied by reduced microglial density and accumulation of uncleared apoptotic cells, linking fructose-driven metabolic rewiring to impaired microglial clearance during neurodevelopment (67) (**Figure 2B**).

Microglial fructose metabolism has also been implicated in diabetes-associated cognitive dysfunction. A study in diabetic mice found that KHK, the rate-limiting enzyme of fructose metabolism, is primarily localized in microglia and upregulated in the hippocampus of these mice (68). Inhibiting KHK reduced microglial activation, restored mitochondrial homeostasis, recovered structural synaptic plasticity, and improved cognitive function. These findings directly link endogenous CNS fructose metabolism in microglia to cognitive dysfunction in a disease model, and importantly, do so independently of systemic dietary fructose exposure.

In our *PNAS* study, we build on this by showing that microglial fructose metabolism influences microglial state and downstream immune responses in glioblastoma (GBM) (69). Our data demonstrates that the GLUT5 transporter is not simply a metabolic factor in microglia, but a pathway that shapes how these cells behave within the tumor microenvironment. Among multiple murine models with GLUT5 knocked out, glioma growth is significantly reduced, with many mice never developing tumors. Additionally, these models show the tumor environment shifting towards stronger immune activation, including increased antigen presentation programs and more robust CD8+ T-cell activation (69) (**Figure 2C**). Moreover, we concluded that this survival benefit depends on adaptive immunity: depleting CD8^+^ T cells abolished the anti-tumor survival benefit observed with GLUT5 loss, supporting the conclusion that GLUT5-driven microglial fructose metabolism is immunosuppressive in GBM. Mechanistically, we found that fructose directly suppressed phagocytosis and subsequent antigen presentation by immune cells, thereby connecting the previous study to ours. While we did not show that the effects were dependent on HK2, we did find that fructose could significantly suppress redox stress in microglia, which is known to drive inflammatory phenotypes (70). At first glance, this appears to contradict the pro-inflammatory framework described above. This likely reflects a combination of enzymatic context and baseline microglial state, where the same substrate may amplify signaling already primed toward inflammation or reinforce an existing suppressive program. The mechanistic basis of this context-dependence remains an open question.

Our results indicate that fructose metabolism drives an immunosuppressive microglial state in GBM, in part by maintaining redox balance and limiting inflammatory signaling. These changes are also accompanied by reduced microglial phagocytosis of apoptotic glioma cells and weaker antigen-specific T-cell responses in co-culture, linking microglial fructose metabolism to microglial shaping of downstream anti-tumor immunity. These findings demonstrate that microglial fructose metabolism shapes microglial behavior across a range of pathological contexts, including neurodevelopment, metabolic disease, and brain tumor immunology. Interestingly, microglial GLUT5 expression decreases in AD mouse models, and whether microglia are exposed to glucose versus fructose has been shown to shape their response to amyloid plaque pathology (71). In AD models, HK2, identified as a potential regulator of fructose-driven microglial suppression, has been shown to regulate amyloid clearance (72). Its deletion promotes ß-amyloid clearance and reduces cognitive impairment, with fructose-6-phosphate identified as a key downstream metabolite in this pathway (72). Most recently, a study found that microglial SLC2A5 deficiency attenuates ischemic brain injury in mice (73). The loss of SLC2A5 reduced fructose-driven activation of PKM2, which is a key glycolytic enzyme that promotes pro-inflammatory microglial reprogramming, and instead shifted microglia toward a neuroprotective state. This further extends the relevance of microglial fructose metabolism and highlights GLUT5 as a potential therapeutic target across CNS disease contexts.

## 7 Evolutionary parallels suggest fructose metabolism is a conserved strategy in specialized fluid compartments

If fructose metabolism is truly functionally specialized for specific cellular contexts, we might expect to find analogous examples elsewhere in biology; cases where a cell type depends on fructose in ways that glucose cannot substitute for. As it turns out, there are, and they provide some important clues into what makes this metabolic strategy unique.

**Figure 3:**
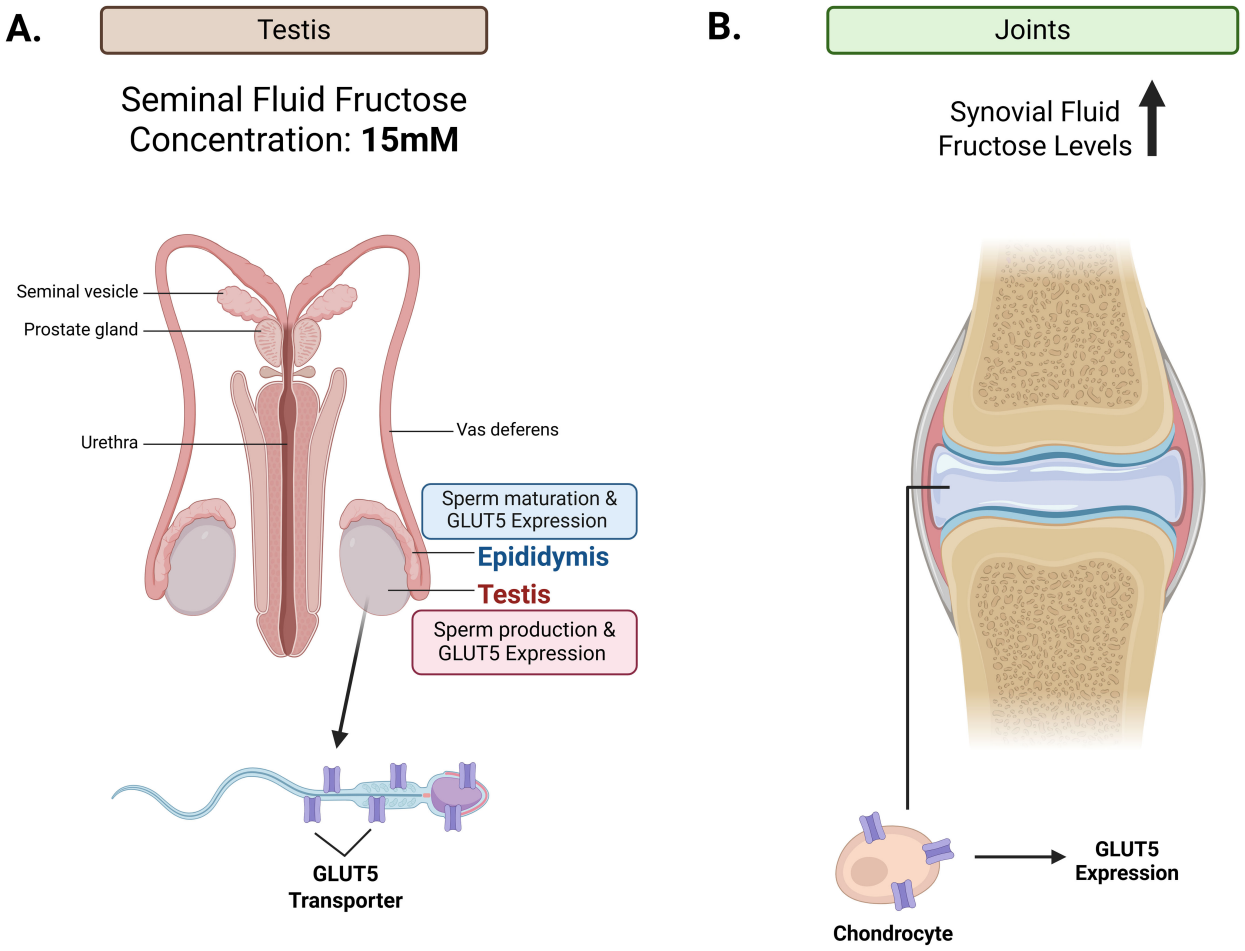
Fructose is enriched in unique fluid sites. In **(A)** the concentration of fructose in seminal fluid is approximately 15mM, and the GLUT5 transporter is constitutively expressed by spermatids. Fructose is the primary metabolic substrate for motility, and a deficiency in GLUT5 severely impairs reproduction in mice. In **(B)** there is fructose in the synovium (concentration unknown), which is increased under inflammation. GEPIA database analysis reveals glut5 expression is highest in synovial tissues compared to all other tissues measured. The consumers of this fructose are largely unknown, although a recent study showed that chondrocytes express high levels of GLUT5 (81).

While fructose transport and metabolism from dietary sources necessitate GLUT5 expression in the gut for its uptake and subsequent metabolism (74), GLUT5 expression is highest in the testis (60). This is because GLUT5 is constitutively expressed on spermatids (75, 76) and is thought to be their main fuel source for motility (77) (**Figure 3A**). Indeed, the mean fructose concentration in semen across several studies is approximately 15 mM (77), well above its levels in any other body fluid. Consistent with this, GLUT5 knockout leads to reproductive deficits and functional impairments in spermatozoa (78). The knockout induced lipid droplet formation, mitochondrial stress, and phenotypes somewhat similar to those observed in GLUT5-deficient microglia in our datasets (69). Examination of tissue vs. malignancy expression of GLUT5 in the GEPIA database reveals that normal synovial tissue is another location of extraordinarily high GLUT5 expression. Indeed, fructose is enriched in synovial fluid and changes during inflammation (79, 80). In another study, chondrocyte stem cells were found to express high levels of GLUT5 and could maintain stem-like phenotypes solely on fructose (81) (**Figure 3B**).

Another consideration is that these tissues have distinct fluid and tissue barriers that separate them from systemic circulation (82, 83). This raises the possibility that fructose metabolism is a broadly shared strategy among resident cells in specialized body fluids, where fructose is locally enriched. Whether this reflects a conserved mechanistic requirement for fructose metabolism in these tissues is an important question that direct cross-tissue comparisons could address. Why evolution converged on this metabolic choice across these tissues remains unclear, but the pattern across tissues is striking and raises questions we believe are worth pursuing. Further understanding of how fructose metabolism influences microglial biology will likely yield critical insights into CNS diseases in which microglia are a central factor.

## 8 Conclusions and thoughts for the future

Our data and broader literature argue that microglial fructose metabolism is not a metabolic quirk; it is a functionally significant feature of CNS biology. GLUT5 is part of the core transcriptional identity of resident microglia; fructose is enriched in the CNS interstitium and may be produced locally through the polyol pathway. Disrupting this axis has profound effects on microglial behavior in contexts ranging from neurodevelopment to brain tumor immunosuppression. The parallels with sperm, chondrocytes, and synovial tissue suggest that this is not a coincidence in CNS biology: fructose metabolism may be a conserved strategy for supporting resident cells in compartments where fructose is locally enriched. Why evolution converged on this solution remains an open question and a genuinely unknown phenomenon. What we do know is that the functional consequences are real and likely extend well beyond the pathologies covered here.

Moreover, we propose three working hypotheses for future research. First, GLUT5-linked fructose metabolism is a functionally significant feature of microglial identity with context-dependent consequences. Second, locally produced CNS fructose provides microglia with a substrate they are uniquely equipped to access via GLUT5, one that may be especially relevant when glucose is limited. Third, the pattern of GLUT5 expression across distinct body fluids reflects a shared metabolic strategy that warrants direct testing. Whether fructose serves as an emergency fuel during the metabolic crisis of brain injury, how it intersects with redox regulation across disease states, and whether GLUT5 can be therapeutically targeted without compromising homeostatic microglial function are all important questions for future work. As microglia have proposed roles in almost any CNS disease, a deeper understanding of brain fructose metabolism, and indeed systemic biology, is essential for future disease treatments.
